# Can response to ADHD medication be predicted?

**DOI:** 10.1007/s00787-025-02650-8

**Published:** 2025-01-29

**Authors:** Maria M. Lilja, Paul Lichtenstein, Eva Serlachius, Jyoti Bhagia, Kerstin Malmberg, Christer Malm, Fabian Lenhard, Linda Halldner

**Affiliations:** 1 Department of Clinical Sciences, Child and Adolescent Psychiatry, Umea University, Umea, Sweden; 2 Department of Medical Epidemiology and Biostatistics, Karolinska Institutet, Stockholm, Sweden; 3 Department of Clinical Sciences, Faculty of Medicine, Section of Child and Adolescent Psychiatry, Lund University, Lund, Sweden; 4 Centre for Psychiatry Research, Department of Clinical Neuroscience, Karolinska Institutet, Stockholm, Sweden; 5 Department of Psychiatry and Psychology, Mayo Clinic, Rochester, MN USA; 6 Centre for Psychiatry Research, Department of Child and Adolescent Research Center, Affiliated to the Department of Clinical Neuroscience, Stockholm, Sweden; 7 Department of Community Medicine and Rehabilitation, Section of Sports Medicine, Umeå School of Sport Sciences, Umea University, Umea, Sweden

**Keywords:** ADHD, Pharmacological treatment, Response, Effect, Predictors

## Abstract

Predictors for the pharmacological effect of ADHD medication in children and adolescents are lacking. This study examined clinically relevant factors in a large (N = 638) prospective cohort reflecting real-world evidence. Children and adolescents aged 6–17 diagnosed with ADHD were evaluated at baseline and three months following ADHD medication initiation. The outcome was measured as a reduction in total SNAP-IV (Swanson Nolan and Pelham teacher and parent rating scale) score at three months compared to baseline. Outcome groups were defined as Responders (≥ 40% reduction), Intermediate Responders (≥ 20 to < 40% reduction), and Non-responders (< 20% reduction). Included independent variables were the Autism Spectrum Questionnaire (ASSQ), the Spence Children’s Anxiety Scale (SCAS), the Pediatric Side Effect Checklist (P-SEC), anthropometrics measures, geographical region, relative age, Children´s Global Assessment Scale (CGAS), Intelligence quotient (IQ), pharmacological treatment initiation month, ADHD symptom severity, ADHD presentation, and psychotic-like experiences. Multinomial logistic regression suggested that ADHD symptom severity, region, relative age, and stating ADHD medication at three-month follow-up were associated with the response outcome group. However, when validating the data with Bootstrap Forest, none of the variables were significant. Thus, in our large naturalistic cohort, we could not identify any clinically relevant factors that reliably predict pharmacological treatment outcomes.

*Trial registration*: NCT02136147.

**Supplementary Information:**

The online version contains supplementary material available at 10.1007/s00787-025-02650-8.

## Introduction

Attention deficit hyperactivity disorder (ADHD) is one the most diagnosed neurodevelopmental disorders and globally affects around 5% of children and adolescents [1, 2]. ADHD is defined as age-inappropriate and impairing inattentiveness, hyperactivity, or impulsivity [3], divided in three predominant presentations: hyperactive-impulsive (ADHD-HI), inattentive (ADHD-I), and combined (ADHD-C).

Insufficiently treated ADHD is associated with adverse long-term life outcomes, such as self-harm, drug abuse [4], academic underachievement [5], accidents and injuries [6], criminality, and psychosocial disability [7]. Based on its efficacy and tolerability in RCTs methylphenidate (MPH) is recommended as the first-line pharmacological treatment for children and adolescents [8].

There is substantial evidence for the short-term efficacy of pharmacological treatment [9] in reducing functional impairment and improving quality of life.

However, studies reporting the highest effect sizes of pharmacological treatment (up to 1.8) are randomized placebo control trials often with highly selected cohorts, which makes it difficult to generalize to clinical settings [10].

Previous research has suggested several factors possibly influencing pharmacological treatment effects such as comorbidity with anxiety [11–15], ADHD symptom severity [11, 14–17], IQ [11, 14, 15], the predominant presentations of ADHD [11, 16, 18–20], and sex [21]. 

Nevertheless, we are still unable to identify what factors predict good outcomes of pharmacological treatment [22]. A reason could be the limited number of prior studies on clinically relevant populations. More representative studies examining naturalistic cohorts focusing on real-life conditions are still lacking [23].

Also, most clinical cohorts suffer from relatively small sample sizes: 518 [11], 207 [14, 24], and 148 [25].

This (N = 638) prospective clinical cohort study aimed to extend the evidence base for factors predicting the efficacy of pharmacological ADHD treatment including the previously never-studied factors: relative age, psychotic-like experiences, blood pressure, heart rate, and month of treatment initiation.

## Methods

### Study population

Participants in our study consisted of those enrolled in the ADHD medication and predictors of treatment outcome (ADAPT) study. The ADAPT study included 638 children and adolescents from July 2014 until May 2021. Participants were recruited from fifteen Swedish Child and Adolescent Psychiatry outpatient units represented from three different regions: Stockholm (13 units), Gotland (one unit), and Västerbotten (one unit). Inclusion criteria were age 6–17 years, established clinical ADHD diagnosis, and about to initiate ADHD medication (methylphenidate, dexamfetamine, lisdexamfetamine, atomoxetine, or guanfacine). Exclusion criteria included prior ADHD medication treatment within three months before study inclusion and a lack of informed consent.

### Study design

Our study is a prospective observational cohort study. Data were collected at the first visit (baseline), one, three, six, and 12-month follow-up. We exclusively used data from the baseline and the three-month follow-up. At every visit, the type and dosage of ADHD medication were registered, and we collected anthropometric data on body weight, height, systolic and diastolic blood pressure, and heart rate. In addition, parents completed questionnaires: the Swanson, Nolan, and Pelham ADHD Rating Scale-version IV(SNAP-IV), the Autism Spectrum Screening Questionnaire (ASSQ), the Spence Children´s Anxiety Scale (SCAS), and the Pediatric Side Effects Checklist (P-SEC).

To obtain further in-depth information on the study participants, we collected information on baseline functioning, measured via Children´s Global Assessment Scale (CGAS) score, as well as patient intelligence quotient (IQ), which was obtained from the National Quality Register for ADHD treatment follow-up (BUSA) described in Supplementary Table S1.

Our ethical permit did not grant access to medical records to obtain missing information on drug selection or titration dosages.

## Measures

### Outcome

We defined treatment effect as ADHD symptom reduction after three months of treatment compared to baseline using the SNAP-IV scale. The SNAP-IV scale has demonstrated good reliability and validity for assessing ADHD symptoms with good-to-excellent internal consistency [26]. Children were divided into three groups defined as responders (≥ 40% SNAP-IV score reduction) [27], intermediate responders (< 40% but ≥ 20%), and non-responders (< 20%).

### Assessments

Anthropometrics: body weight, height, systolic and diastolic blood pressure, and heart rate.

### Questionnaires

To assess the predominant presentation of ADHD symptoms we used the SNAP-IV questionnaire´s predefined subscales. In addition, we evaluated Oppositional Defiant Disorder symptoms [28].

To identify children with graded autism symptoms, we used the baseline total score from the validated ASSQ questionnaire [29].

To assess anxiety symptoms, we used the baseline total score from the SCAS-P questionnaire [30].

To evaluate psychiatric and somatic symptoms at the study start before pharmacological treatment initiation, we used the baseline total score from the P-SEC scale [31].

### Other variables

To evaluate relative age, the tertile distribution of birth month was assessed: First tertile (January-April), second tertile (May–August), and third tertile (September-December)[32].

To examine the influence of the pharmacological treatment initiation month, the prescription months were categorized into tertiles, as described in the previous section.

IQ levels were assessed ranging from ≤ 69 to ≥ 131 and consolidated into four groups: Above average (IQ ≥ 108), Average (IQ 93–107), Below average (IQ ≤ 92), and Difficult to assess.

To assess psychotic-like experiences, we used a separate single question: Have you ever heard voices or sounds that nobody else can hear? [33]. The answer ‘yes definitely’ or ‘maybe’, was considered as a psychotic-like experience. The answer ‘no’ was defined as the absence of psychotic-like experiences.

To capture the functional ability, we included the CGAS score, categorized into five groups according to Shaffer et al. [34].

To assess regional differences, the Stockholm, Västerbotten, and Gotland regions were compared.

Current ADHD medication at three months included monotherapy or combinations.

Supplementary Table S2 provides a summary of all measurements.

### Missing data

A substantial amount of the variables (67%) suffered from any missing data, ranging from 0.7% (diastolic blood pressure) to 66.3% (body weight). To maximize information density, the number of complete cases was balanced with the number of variables until we had 89% complete cases. This procedure left us with 13 variables for the final model. However, separate unadjusted Multinomial logistic regressions were also performed for all 22 variables.

A more stringent criterion was applied when defining outcome groups. Initially, individuals lacking the total score for any SNAP-IV scale (N = 216) were excluded, resulting in 422 individuals. Additionally, individuals with > 2 missing items on the SNAP-IV scales were excluded in a two-step procedure, first for SNAP-IV at baseline and second for SNAP-IV at the three-month follow-up, resulting in a final cohort comprising 419 individuals. (Two females and one male were excluded). Consequently, no participant in the final cohort exhibited more than two missing items on either of the SNAP-IV scales.

Despite these procedures, the number of subjects included in the different regression analyses varied somewhat.

### Statistical analyses

First, to test for differences in baseline characteristics between our outcome groups, and individuals with a completely missing SNAP-IV scale at baseline and follow-up, we used the Kruskal–Wallis one-way analysis of variance test for the rating scales: ASSQ, SNAP-IV, P-SEC, and SCAS. One-way ANOVA tests were performed for the continuous anthropometric variables, and age. For categorical variables, (tertiles of birth, tertiles of pharmacological treatment initiation month, regions, pharmacological treatment, psychotic-like experiences, sex, IQ, and CGAS) we conducted the Chi-Square test of independence.

Second, to evaluate predictive factors unadjusted multinomial logistic regressions for each of the 22 variables were conducted.

**Fig. 1:**
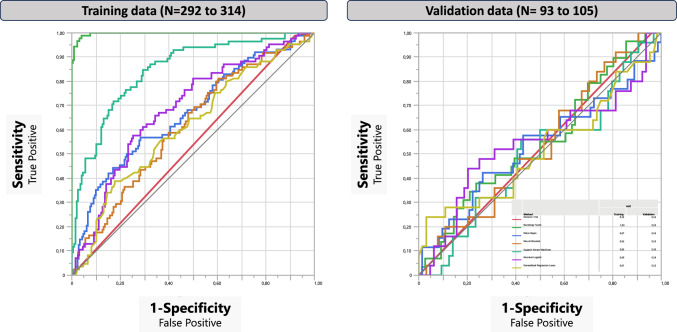
The Machine Learning model screening in the Training and Validation dataset including the 13 selected variables showing the best fit for Bootstrap Forest

Third, to explore patterns of independent variables as predictors, we applied machine learning techniques to our final model with the selected 13 variables. [35]. Random forest with 1000 decision trees first ranked all included predictor variables by their contribution to the model. Variables contributing with > 5% were entered into Model Screening, comparing the performance of seven different machine learning methods (Fig. 1). Models were created using 75% of the data for training and 25% for validation. The machine learning model with the highest R^2^ was chosen for further analysis.

Fourth, we performed subanalyses to assess if ADHD medication substances differed between the outcome groups and sensitivity analyses on the cohort within the Västerbotten Region to affirm the robustness of our primary analyses, despite missing data. Data collection in the Västerbotten Region was overseen by a designated research nurse, minimizing instances of missing data. An overview of statistical analyses is provided in Supplementary Table S3.

Statistical analysis was performed using SPSS software (Version 28.1.1.0, IBS Corp., Armonk, NY, USA) and JMP software (Version 16.2, SAS Institute, Cary, NC, USA). All tests were two-sided, and significance was set at P < 0.05.

## Results

### Descriptive study population characteristics

A total of 638 children and adolescents were enrolled in the study. The mean age was 11.6 years old, the median age was 12 years old, and 63.5% (N = 405) were male. Geographically, 82% (N = 522) were from Stockholm, 15% (N = 97) from Västerbotten, and 3% (N = 19) from Gotland. During the three-month follow-up, 216 participants were lost to follow-up, and three were excluded due to missing data on the SNAP-IV scale, as described in the Missing data analysis section. Consequently, the final cohort consisted of 419 participants. Descriptive baseline characteristics are presented in Tables 1 and 2.

**Table 1:** Descriptive Baseline Characteristics for Non-responders, Intermediate Responders, Responders, and Excluded. Presentation of Continuous variables

Variable	Non-Responders	Intermediate responders	Responders	Excluded	P
Age (years)	164	118	137	219	
	11.6 (11.1–12.1)	11.1 (10.5–11.7)	11.7 (11.2–12.2)	11.8 (11.4–12.3)	0.25
Body weight (kg)	161	118	136	186	
	47.6 (44.6–50.6)	48.0 (44.2–51.7)	48.8 (45.8–51.7)	48.3 (45.6–51.0)	0.96
Body height (cm)	162	118	136	186	
	151 (149–154)	151 (148–155)	154 (151–156)	153 (150–155)	0.63
BMI (kg/m^2^)	161	118	136	186	
	20.0 (19.2–20.7)	20.0 (19.1–20.9)	20.0 (19.3–20.8)	19.9 (19.3–20.5)	0.99
Resting HR (bpm)	155	116	133	182	
	75.6 (73.9–77.3)	76.4 (74.4–78.4)	74.6 (72.7–76.5)	74.9 (73.2–76.5)	0.55
Diastolic BP (mmHg)	162	118	136	185	
	66.0 (64.8–67.1)	65.2 (63.7–66.6)	65.9 (64.5–67.4)	66.5 (65.4–67.6)	0.56
Systolic BP (mmHg)	161	118	136	184	
	110 (108–113)	110 (108–112)	111 (109–113)	108 (106–110)	0.07
ASSQ^a^	159	117	135	190	
	10 (10–12)	10 (10–13)	9 (8–12)	10 (9–13)	0.23
SCAS-P^a^	159	115	135	190	
	19 (18–24)	19 (18–23)	23 (21–26)	21 (21–25)	0.55
P-SEC^a^	163	118	132	192	
	18 (17–21)	17 (17–22)	17 (16–21)	21 (20–24)	< 0.01
SNAP-IV^a^	164	118	137	183	
	45 (43–51)	54 (47–60)	51 (47–46)	49 (47–55)	< 0.01
SNAP hyperactivity^a^	164	118	137	183	
	13 (12–16)	17 (16–20)	14 (13–17)	14 (14–17)	0.02
SNAP inattention^a^	164	118	137	183	
	18 (18–20)	19 (19–21)	21 (21–23)	19 (19–21)	< 0.01
SNAP ODD^a^	164	118	137	183	
	10 (9–12)	10 (9–12)	10 (8–12)	12 (12–14)	0.13
SNAP combined^a^	164	118	137	183	
	30 (29–32)	36 (32–36)	35 (32–36)	32 (30–33)	< 0.01
CGAS^a^	130	93	102	0	
	51 (51–53)	51 (50–55)	51 (50–54)	0 (0–0)	0.89

N and Mean or ^a^Median (95% CI). Median CI by 1000 Boostrap samples. P: ANOVA or Chi^2^ by Kruskal–Wallis for non-parametric data

**Table 2:** Descriptive baseline characteristics for Non-responders, Intermediate responders, Responders, and Excluded. Presentation of categorical variables

Variable	Non-responders	Intermediate responders	Responders	Excluded	Total	P
Sex						0.77
Male	108 (17%)	77 (12%)	83 (13%)	137 (21%)	405 (63%)	
Female	56 (9%)	41 (6%)	54 (8%)	82 (13%)	233 (37%)	
Birthmonth per tertile						0.18
Jan to Apr	61 (10%)	32 (5%)	48 (8%)	72 (11%)	213 (33%)	
May to Aug	40 (6%)	45 (7%)	40 (6%)	77 (12%)	202 (32%)	
Sep to Dec	63 (10%)	41 (6%)	49 (8%)	70 (11%)	223 (35%)	
Treatment initiation month per tertil						0.40
Jan to Apr	44 (27%)	35 (30%)	47 (34%)	83 (38%)	209 (33%)	
May to Aug	78 (48%)	55 (47%)	59 (43%)	92 (42%)	284 (45%)	
Sep to Dec	42 (26%)	28 (24%)	31 (23%)	44 (20%)	145 (23%)	
Psychotic-like experience						0.23
No	121 (21%)	98 (17%)	105 (18%)	134 (23%)	458 (80%)	
Yes or Maybe	33 (6%)	16 (3%)	25 (4%)	41 (7%)	115 (20%)	
CGAS score at study by categories						0.33
Unable to function (21–30)	0 (0%)	0 (0%)	1 (0%)	0 (0%)	1 (0.3%)	
Major impairment (31–40)	3 (1%)	8 (2%)	5 (2%)	0 (0%)	16 (4.9%)	
Moderate degree of interference (41–50)	54 (17%)	38 (12%)	38 (12%)	0 (0%)	130 (40%)	
Variable functioning (51–60)	68 (21%)	43 (13%)	50 (15%)	0 (0%)	161 (50%)	
Some difficulty (61–70)	5 (2%)	4 (1%)	8 (2%)	0 (0%)	17 (5.2%)	
IQ at baseline						0.69
Difficult to assess	3 (1%)	5 (2%)	5 (2%)	2 (1%)	15 (4.7%)	
Above average (IQ ≥ 108)	21 (7%)	11 (3%)	10 (3%)	19 (6%)	61 (19%)	
Average (IQ 93 to107)	41 (13%)	23 (7%)	33 (10%)	44 (14%)	141 (44%)	
Below average (IQ ≤ 92)	25 (8%)	23 (7%)	29 (9%)	26 (8%)	103 (32%)	

Distribution between categories N (% of total). P: Chi^2^ Likelihood ratio for categories

### Multinomial logistic regression

Unadjusted logistic regression analysis for each of the 22 variables revealed that high total SNAP scores, SNAP inattention scores, SNAP hyperactivity/impulsivity scores, and SNAP combined scores, as well as region, relative age, and stating ADHD medication at three-month follow-up were significantly associated with outcome group. Psuedo R-square (McFadden) values for the unadjusted multinomial regression models were notably low, ranging from 0.007 for tertiles of birth to 0.018 for inattention scores. A complete overview of the unadjusted multinomial logistic regressions is provided in Table 3. There were no significant interactions of the independent variables in the model.

**Table 3:** Unadjusted Multinominal Logistic Regression for all 22 variables. Presented per variable

Term	Model Chi^2^	P		vs non-responder	Estimate		SE	Chi^2^	P	OR (95% CI)
ASSQ	2.12	0.35		Responder	− 0.01		0.02	0.68	0.41	0.98 (0.95–1.01)
				Intermediate	0.01		0.01	0.51	0.47	1.01 (0.98–1.04)
P-SEC	2.2	0.33		Responder	− 0.01		0.01	1.73	0.19	0.98 (0.96–1.00)
				Intermediate	0.00		0.01	0.00	0.97	1.00 (0.98–1.02)
SNAP-IV	8.39	0.02		Responder	0.01		0.01	5.13	0.02*	1.01 (1.00–1.02)
				Intermediate	0.02		0.01	6.65	0.01*	1.01 (1.00–1.03)
Resting HR (bpm)	1.73	0.42		Responder	− 0.01		0.01	0.64	0.42	0.99 (0.97–1.01)
				Intermediate	0.01		0.01	0.33	0.56	1 .00 (0.98–1.02)
SBP (mmHg)	0.92	0.63		Responder	0.00		0.01	0.25	0.61	1.00 (0.98–1.02)
				Intermediate	− 0.01		0.01	0.26	0.61	0.99 (0.97–1.01)
SCAS-P	1.65	0.43		Responder	0.00		0.01	0.29	0.59	1 .00 (0.98–1.02)
				Intermediate	− 0.01		0.01	0.66	0.42	0.99 (0.97–1.01)
DBP (mmHg)	0.84	0.66		Responder	0.00		0.01	0.00	1.00	0.99 (0.97–1.02)
				Intermediate	− 0.01		0.02	0.68	0.41	0.98 (0.95–1.01)
Age (years)	2.57	0.28		Responder	0.01		0.04	0.15	0.70	1.01 (0.94–1.09)
				Intermediate	− 0.05		0.04	1.51	0.22	0.95 (0.88–1.02)
Birthmonth per Tertile	6.82	0.15								
Jan-April vs. Sep-Dec				Responder	− 0.08		0.16	0.22	0.64	0.92 (0.67–1.27)
May-Aug vs. Sep-Dec				Responder	0.16		0.17	0.88	0.35	1.17 (0.83–1.65)
Jan-Apr vs. Sep-Dec				Intermediate	− 0.33		0.18	3.44	0.06	0.72 (0.51–1.01)
May-Aug vs.Sep-Dec				Intermediate	0.44		0.18	6.20	0.01*	1.54 (1.09–2.18)
Initiation of adhd medication	2.02	0.73								
Jan-April/Sep-Dec				Responder	0.24		0.17	1.95	0.16	1.26 (0.90–1.77)
May-Aug/Sep-Dec				Responder	− 0.11		0.16	0.47	0.49	0.89 (0.66–1.22)
Jan-Apr/Sep-Dec				Intermediate	0.10		0.18	0.30	0.58	1.10 (0.77–1.57)
May-Aug/Sep-Dec				Intermediate	− 0.02		0.16	0.02	0.89	0.97 (0.71–1.34)
Region (Västerbotten as reference)	10.0	0.04								
Gotland vs. Västerbotten				Responder	0.88		0.40	4.88	0.03*	2.40 (1.10–5.23)
Stockholm vs. Västerbotten				Responder	− 0.49		0.23	4.60	0.03*	0.61 (0.39–0.95)
Gotland vs. Västerbotten				Intermediate	− 0.29		0.59	0.25	0.62	0.74 (0.23–2.35)
Stockholm vs. Västerbotten				Intermediate	0.02		0.31	0.00	0.94	1.02 (0.55–1.89)
Sex (Boy as reference)	1.01	0.60								
				Responder	− 0.11		0.12	0.89	0.34	0.89 (0.70–1.12)
				Intermediate	− 0.01		0.13	0.01	0.92	0.98 (0.76–1.26)
Psychotic-like experiences (No as reference)	2.50	0.25		Responder	0.07		0.15	0.21	0.65	1.07 (0.80–1.43)
				Intermediate	0.26		0.17	2.37	0.12	1.29 (0.93–1.79)
ADHD medication at 3 months (Yes as reference)	7.91	0.02		Responder	− 0.68		0.40	2.97	0.09	0.51 (0.23–1.10)
				Intermediate	− 1.21		0.48	6.30	0.01*	0.30 (0.12–0.77)
BMI	0.01	0.99		Responder	0.00		0.03	0.01	0.92	1.00 (0.95–1.05)
				Intermediate	0.00		0.03	0.00	0.95	1.00 (0.95–1.05)
Body height (cm)	1.50	0.47		Responder	0.01		0.01	1.27	0.26	1.00 (0.99–1.02)
				Intermediate	0.00		0.01	0.00	0.95	1.00 (0.98–1.01)
Body weight (kg)	0.03	0.87		Responder	0.00		0.01	0.26	0.61	1.00 (0.99–1.01)
				Intermediate	0.00		0.01	0.02	0.88	1.00 (0.98–1.01)
SNAP inattention	16.2	< 0.01		Responder	0.09		0.02	15.36	< 0.01*	1.09 (1.04–1.14)
				Intermediate	0.04		0.02	3.31	0.07	1.04 (0.99–1.09)
SNAP hyperactivity/impulsivity	8.60	< 0.01		Responder	0.02		0.01	1.97	0.16	1.02 (0.99–1.05)
				Intermediate	0.05		0.02	8.37	< 0.01*	1.04 (1.01–1.08)
SNAP comb (inatt and hyper/impuls)	11.9	< 0.01		Responder	0.03		0.01	8.28	< 0.01*	1.03 (1.00–1.05)
				Intermediate	0.03		0.01	8.45	< 0.01*	1.03 (1.01–1.05)
SNAP ODD	2.10	0.35		Responder	0.01		0.02	0.56	0.45	1.01 (0.97–1.04)
				Intermediate	0.03		0.02	2.08	0.15	1.02 (0.99–1.06)
CGAS	0.52	0.77		Responder	0.00		0.02	0.01	0.92	1.00 (0.96–1.04)
				Intermediate	− 0.01		0.02	0.36	0.55	0.98 (0.94–1.02)
IQ level	4.85	0.56								
Above average (IQ > 108) vs. Difficult to assess			Responder		0.57	0.67		0.74	0.39	1.77 (0.47–6.55)
Average (92–108) vs. Above average (IQ > 108)			Responder		− 0.02	0.51		0.00	0.97	0.98 (0.36–2.65)
Below average (IQ < 92) vs. Average (92–108)			Responder		0.37	0.36		1.03	0.31	1.44 (0.71–2.91)
Above average (IQ > 108) vs. Difficult to assess			Intermediate		− 0.34	0.66		0.27	0.60	0.70 (0.19–2.58)
Average (92–108) vs. Above average (IQ > 108)			Intermediate		0.03	0.57		0.00	0.96	1.02 (0.33–3.14)
Below average (IQ < 92) vs. Average (92–108)			Intermediate		0.49	0.39		1.62	0.20	1.64 (0.76–3.51)

Estimate: the coefficient estimate represents the change in the log odds of the outcome variable associated with a one-unit change in the predictor variable. Positive values suggest an increase in the odds of the outcome, negative values suggest a decrease. The magnitude of the coefficient indicates the strength of the effect. Chi^2^ by Wald test. Non-responder set as reference. *Significant *P* < 0.05

### Machine learning model screening

**Fig. 2:**
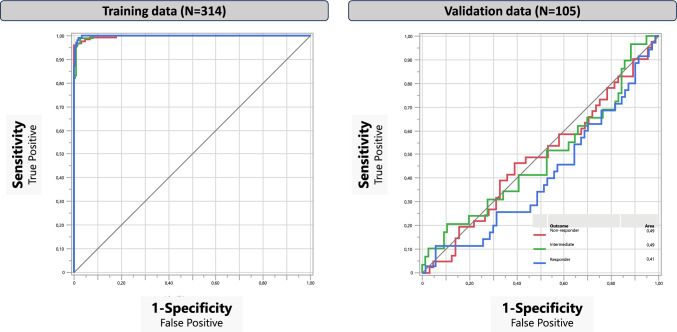
Bootstrap Forest Training and Validation Models including the selected 13 variables displaying outcome groups. The contribution of each variable is displayed in Table 4

When Machine Learning model screening was performed including the thirteen variables,

Bootstrap Forest showed the most robust model in the training dataset and was chosen for detailed analysis. In the validation dataset none of the Machine Learning Models were significant (Fig. 1). Validating the model on the 25% independent data (not part of the training model), resulted in an R^2^ = − 0.05, AUC = 0.48, and Misclassification Rate = 64% (Fig. 2).

**Table 4:** Bootstrap Forest training model. Variable contribution for each of the 13 selected variables

Term	G^2^	Portion (%)
ASSQ	32	12
P-SEC	31	12
SNAP-IV	29	11
Resting HR (bpm)	26	10
Resting systolic BP (mmHg)	25	10
SCAS-P	23	9
Resting diastolic BP (mmHg)	22	8
Age (years)	19	7
Birthmonth per tertile	14	5
Initiation of adhd mediciation by tertil	13	5
Clinic	13	5
Sex	9	3
Psychotic-like experiences	6	2

G^2^: the likelihood ratio goodness-of-fit test (likelihood ratio Chi^2^): it compares the loglikelihood for the independence model to the loglikelihood without the assumption of independence

### Sub-analysis

At the three-month follow-up, 203 individuals reported monotherapy with methylphenidate, 54 with lisdexamfetamine, 21 with atomoxetine, and three with guanfacine. None reported using dexamphetamine. Three individuals received combination treatment: two with methylphenidate and atomoxetine and one with lisdexamphetamine and guanfacine. Additionally, 25 participants declared no medication. Information on current medication was missing for 110 participants. Statistical analysis revealed no significant differences in reported medication between the outcome groups (Pearson Chi-Square P = 0.29).

### Sensitivity analysis

The sensitivity analysis on the Västerbotten subcohort did not reveal any significant differences in the results compared to the same analyses on the full dataset.

## Discussion

In this study, we aimed to identify clinically relevant predictive factors for pharmacological ADHD treatment effects in children and adolescents in a clinical setting. When performing unadjusted multinomial logistic regression without validation, baseline higher SNAP scores for inattention, combined, and the total score, and being treated in the Gotland region compared to the Västerbotten region as well as being treated in the Västerbotten region compared to the Stockholm region, increased the odds of being a responder compared to non-responders. Baseline higher SNAP scores for hyperactivity/impulsivity, combined, and the total score, as well as stating ADHD medication at three months, and being born in the second tertile compared to the third tertile, increased the odds of being an intermediate responder compared to non-responders. However, none of the significant variables could explain more than 1.8% of the variation in the model. Consequently, even though crude estimates were significant, we could not identify any variables predicting pharmacological treatment effect.

Our findings align with prior research indicating that more severe ADHD symptoms tend to correlate with improved treatment outcomes [16]. In contrast, some studies suggested that more severe ADHD symptoms might predict poorer treatment outcomes [11, 14]. The discrepancies in findings could be attributed to variations in measurement criteria and defining outcomes. We applied a strict definition of responders (≥ 40% SNAP score reduction) [36]. One-third (33%) in our cohort were classified as responders, which in comparison to other studies, is a low number. [11, 14]. However, reducing the responder definition to ≥ 30% did not yield significant changes in the results. Neither did analyzing SNAP-IV scores as a continuous variable. Also, there is no accepted definition of clinically significant treatment response [10].

In our data set, the Gotland Region demonstrated a significantly higher number of responders compared to the other participating regions. Nevertheless, only 19 participants from Gotland were included in our study. Consequently, drawing any definitive conclusions from this result is not feasible.

To the best of our knowledge, our study is the first to examine blood pressure, heart rate, relative age, pharmacological treatment initiation month, and psychotic-like experiences in the context of response rate of ADHD medication. Blood pressure and heart rate are closely intertwined with the sympathetic and parasympathetic nervous systems which are affected by many psychiatric disorders [37]. Psychotic-like experiences has been shown to strongly correlate with clinical psychotic disorder [33], which might indicate severe psychiatric illness. The relative age effect refers to the phenomenon where individuals born closer to the cutoff date for school start demonstrating advantages over their younger peers. Studies have shown that the youngest children in a school class are more likely to be diagnosed with ADHD [38, 39] or receive ADHD medication [40]. Thus, we hypothesized relative age might affect our outcome. Surprisingly, in our study, tertiles of birth month were evenly distributed throughout the year and had only a minor effect on treatment outcome.

To further investigate the predictive potential of our variables, we applied Machine Learning algorithms to our dataset [41]. The ROC curve for the Boostrap Forest model was significant within the training dataset but could not be replicated in the independent validation dataset. This means that none of our variables, including those identified as significant through Multinomial Logistic Regression analyses, could predict treatment outcomes when subjected to novel data.

Our results accord with earlier studies showing that despite extensive research, the evidence regarding factors associated with outcome of pharmacological treatment remains inconclusive.

### Strengths and limitations

This study’s primary strength is that it expands the scope of current research on factors linked to pharmacological treatment outcome through its contribution with an extensive sample size of ADHD patients derived from a representative clinical cohort, which mirrors real-world evidence. We recruited children and adolescents from three Swedish regions and introduced clinically relevant predictive factors, some never studied before.

Moreover, we performed both conventional Multinomial Logistic Regression and Machine learning techniques to ensure the robustness of our findings. Validation with Multinomial Logistic Regression and Boostrap Forest resulted in identical results, further bolstering our conclusion.

Our study underscores the challenges with incomplete data, frequently encountered in clinical research. This was particularly evident regarding information on current ADHD medication. A considerable number of individuals lacked information on ADHD medication at three months, categorizing them as missing cases. However, according to local clinical guidelines, children discontinuing pharmacological treatment were excluded from the psychiatry units. Thus, presumably, all children in the cohort, except for those stating No medication, had medication at three months. This likely resulted in an underestimation of individuals with ADHD medication, potentially influencing our models.

Varying attrition rates and loss to follow-up might introduce a bias. However, there were no significant differences in baseline characteristics between children with and without a three-month follow-up. Importantly, when we performed sensitivity analyses with more complete data, all our results were sustained, indicating the robustness of our dataset.

Unfortunately, we lacked information on medication compliance, a factor that could influence response to treatment. Neither could we evaluate the influence of medication dosage due to insufficiently registered data. Also, the Ethical permit did not allow access to medical records. Studies have demonstrated that increasing the dosage of stimulant medication enhances pharmacological efficacy [42]. However, Vallejo-Valdivielso et al. did not find a difference in MPH dose between responders and non-responders [11].

Information on eligible patients declining participation was lacking, thus participation rate could not be calculated. Lastly, the questionnaires in the study are all parent-rated, which could have distorted presenting symptoms in our cohort. Including clinician- and teacher-ratings would have been beneficial.

### Contribution and interpretation

Our negative findings are important as an increasing number of children and adolescents are treated with ADHD medication, even though it is not well elucidated if a child will benefit from treatment.

Our results support the compiled evidence that to date, distinct predictors for the treatment effect of ADHD medication have not yet been revealed [22].

There are several possible explanations for the challenge of finding predictors of treatment outcomes.

The inconsistency across studies giving rise to precarious comparisons, implies that the factors influencing pharmacological treatment effects may be as multifaceted as the ADHD diagnosis itself. Further, we might not measure the right things [43]. Despite the use of validated questionnaires, our findings indicate that the tools used may not fully capture the fundamental challenges faced by children and adolescents with ADHD.

Another potential explanation may be that the medication primarily addresses factors beyond the DSM-5 diagnostic symptoms of ADHD, which are currently not measurable.

Although more studies on the influence of external factors, such as e.g. school environment, and traumatic experiences [44] are needed, it may be more successful in addressing underlying transdiagnostic neurophysiological [45]and biological features [46]as possible predictors of ADHD medication response.

## Conclusion

In our large nationwide naturalistic cohort, we found no biological, clinical, or cognitive factors reliably predicting pharmacological treatment outcomes in children and adolescents. To date, clear predictors of pharmacological ADHD treatment effect remain elusive. Given these circumstances, it is reasonable to consider a new perspective regarding which child would benefit the most from pharmacological treatment for ADHD. From a clinical perspective, we raise ethical concerns about withholding pharmacological treatment to children and adolescents who are not yet diagnosed with ADHD or do not meet diagnostic criteria but exhibit severe functional impairments. Conversely, a diagnosis of ADHD alone may not automatically warrant medication. Future work is required to examine whether a shift toward individualized pharmacological treatment, in contrast to a one-size-fits-all approach, is indicated.

## Supplementary Information

Below is the link to the electronic supplementary material.

Supplementary file1 (DOCX 28 KB)

Supplementary file2 (DOCX 34 KB)

## Data Availability

Due to legal reasons and the ethical permit for the study, the data that support the findings of this study are not publicly available. The ethics approval was obtained for public sharing and data presentation on the group level only. The data can only be used for the approved research. The data are accessible on reasonable request from the corresponding authors. Original data are deposited at Karolinska Institutet.

## References

[1] Sayal K, Prasad V, Daley D et al (2018) ADHD in children and young people: prevalence, care pathways, and service provision. Lancet Psychiatry 5:175–186. 10.1016/S2215-0366(17)30167-0 29033005 10.1016/S2215-0366(17)30167-0

[2] Polanczyk GV, Salum GA, Sugaya LS et al (2015) Annual research review: A meta-analysis of the worldwide prevalence of mental disorders in children and adolescents. J Child Psychol Psychiatry 56:345–365. 10.1111/jcpp.12381 25649325 10.1111/jcpp.12381

[3] Association AP (2013) Diagnostic and statistical manual of mental disorders, 5th edn (DSM 5). American Psychiatric Association, Washington, DC

[4] Thapar A, Cooper M (2016) Attention deficit hyperactivity disorder. Lancet 387:1240–1250. 10.1016/S0140-6736(15)00238-X 26386541 10.1016/S0140-6736(15)00238-X

[5] Arnold LE, Hodgkins P, Kahle J et al (2020) Long-Term Outcomes of ADHD: Academic Achievement and Performance. J Atten Disord 24:73–85. 10.1177/1087054714566076 25583985 10.1177/1087054714566076

[6] Vaa T (2014) ADHD and relative risk of accidents in road traffic: a meta-analysis. Accid Anal Prev 62:415–425. 10.1016/j.aap.2013.10.003 24238842 10.1016/j.aap.2013.10.003

[7] Faraone SV, Asherson P, Banaschewski T et al (2015) Attention-deficit/hyperactivity disorder. Nat Rev Dis Primers 1:15020. 10.1038/nrdp.2015.20 27189265 10.1038/nrdp.2015.20

[8] Cortese S, Adamo N, Del Giovane C et al (2018) Comparative efficacy and tolerability of medications for attention-deficit hyperactivity disorder in children, adolescents, and adults: a systematic review and network meta-analysis. Lancet Psychiatry 5:727–738. 10.1016/S2215-0366(18)30269-4 30097390 10.1016/S2215-0366(18)30269-4 PMC6109107

[9] Catala-Lopez F, Hutton B, Nunez-Beltran A et al (2017) The pharmacological and non-pharmacological treatment of attention deficit hyperactivity disorder in children and adolescents: A systematic review with network meta-analyses of randomised trials. PLoS ONE 12:e0180355. 10.1371/journal.pone.0180355 28700715 10.1371/journal.pone.0180355 PMC5507500

[10] Storebo OJ, Ramstad E, Krogh HB et al (2015) Methylphenidate for children and adolescents with attention deficit hyperactivity disorder (ADHD). Cochrane Database Syst Rev:CD009885. 10.1002/14651858.CD009885.pub2 10.1002/14651858.CD009885.pub2 PMC8763351 26599576

[11] Vallejo-Valdivielso M, de Castro-Manglano P, Diez-Suarez A et al (2019) Clinical and Neuropsychological Predictors of Methylphenidate Response in Children and Adolescents with ADHD: A Naturalistic Follow-up Study in a Spanish Sample. Clin Pract Epidemiol Ment Health 15:160–171. 10.2174/1745017901915010160 32174998 10.2174/1745017901915010160 PMC7040471

[12] Reale L, Bartoli B, Cartabia M et al (2017) Comorbidity prevalence and treatment outcome in children and adolescents with ADHD. Eur Child Adolesc Psychiatry 26:1443–1457. 10.1007/s00787-017-1005-z 28527021 10.1007/s00787-017-1005-z

[13] Chan MH, Leung PW, Ho TP et al (2017) Are psychiatric comorbidities and associated cognitive functions related to treatment response to methylphenidate in boys with attention-deficit/hyperactivity disorder? Neuropsychiatr Dis Treat 13:1071–1080. 10.2147/NDT.S128086 28442911 10.2147/NDT.S128086 PMC5396959

[14] Kaalund-Brok K, Houmann TB, Hebsgaard MB et al (2021) Outcomes of a 12-week ecologically valid observational study of first treatment with methylphenidate in a representative clinical sample of drug naive children with ADHD. PLoS ONE 16:e0253727. 10.1371/journal.pone.0253727 34673771 10.1371/journal.pone.0253727 PMC8530346

[15] van der Oord S, Prins PJ, Oosterlaan J, Emmelkamp PM (2008) Treatment of attention deficit hyperactivity disorder in children. Predictors of treatment outcome. Eur Child Adolesc Psychiatry 17:73–81. 10.1007/s00787-007-0638-8 17876505 10.1007/s00787-007-0638-8

[16] Park S, Kim BN, Cho SC et al (2013) Baseline severity of parent-perceived inattentiveness is predictive of the difference between subjective and objective methylphenidate responses in children with attention-deficit/hyperactivity disorder. J Child Adolesc Psychopharmacol 23:410–414. 10.1089/cap.2013.0031 23952188 10.1089/cap.2013.0031

[17] Arns M, Vollebregt MA, Palmer D et al (2018) Electroencephalographic biomarkers as predictors of methylphenidate response in attention-deficit/hyperactivity disorder. Eur Neuropsychopharmacol 28:881–891. 10.1016/j.euroneuro.2018.06.002 29937325 10.1016/j.euroneuro.2018.06.002

[18] Gorman EB, Klorman R, Thatcher JE, Borgstedt AD (2006) Effects of methylphenidate on subtypes of attention-deficit/hyperactivity disorder. J Am Acad Child Adolesc Psychiatry 45:808–816. 10.1097/01.chi.0000214191.57993.dd 16832317 10.1097/01.chi.0000214191.57993.dd

[19] Beery SH, Quay HC, Pelham WE Jr (2017) Differential Response to Methylphenidate in Inattentive and Combined Subtype ADHD. J Atten Disord 21:62–70. 10.1177/1087054712469256 23283758 10.1177/1087054712469256

[20] Chazan R, Borowski C, Pianca T et al (2011) Do phenotypic characteristics, parental psychopathology, family functioning, and environmental stressors have a role in the response to methylphenidate in children with attention-deficit/hyperactivity disorder? A naturalistic study from a developing country. J Clin Psychopharmacol 31:309–317. 10.1097/JCP.0b013e318217b4df 21508864 10.1097/JCP.0b013e318217b4df

[21] Kok FM, Groen Y, Fuermaier ABM, Tucha O (2020) The female side of pharmacotherapy for ADHD-A systematic literature review. PLoS ONE 15:e0239257. 10.1371/journal.pone.0239257 32946507 10.1371/journal.pone.0239257 PMC7500607

[22] Pagnier M (2023) Predicting the Response of Children and Adolescents With ADHD to Methylphenidate: A Systematic Review. J Atten Disord 27:1377–1392. 10.1177/10870547231177234 37243373 10.1177/10870547231177234

[23] Mechler K, Banaschewski T, Hohmann S, Hage A (2022) Evidence-based pharmacological treatment options for ADHD in children and adolescents. Pharmacol Ther 230:107940. 10.1016/j.pharmthera.2021.107940 34174276 10.1016/j.pharmthera.2021.107940

[24] D'Aiello B, Di Vara S, De Rossi P et al (2022) Moderators and Other Predictors of Methylphenidate Response in Children and Adolescents with ADHD. Int J Environ Res Public Health 19. 10.3390/ijerph19031640 10.3390/ijerph19031640 PMC8834961 35162663

[25] Houmann TB, Kaalund-Brok K, Clemmensen L et al (2023) Early treatment response as predictor of long-term outcome in a clinical cohort of children with ADHD. Eur Child Adolesc Psychiatry. 10.1007/s00787-023-02158-z 36795232 10.1007/s00787-023-02158-z PMC10869385

[26] Bussing R, Fernandez M, Harwood M et al (2008) Parent and teacher SNAP-IV ratings of attention deficit hyperactivity disorder symptoms: psychometric properties and normative ratings from a school district sample. Assessment 15:317–328. 10.1177/1073191107313888 18310593 10.1177/1073191107313888 PMC3623293

[27] Newcorn JH, Kratochvil CJ, Allen AJ et al (2008) Atomoxetine and osmotically released methylphenidate for the treatment of attention deficit hyperactivity disorder: acute comparison and differential response. Am J Psychiatry 165:721–730. 10.1176/appi.ajp.2007.05091676 18281409 10.1176/appi.ajp.2007.05091676

[28] Hall CL, Guo B, Valentine AZ et al (2020) The Validity of the SNAP-IV in Children Displaying ADHD Symptoms. Assessment 27:1258–1271. 10.1177/1073191119842255 30991820 10.1177/1073191119842255

[29] Mattila ML, Jussila K, Linna SL et al (2012) Validation of the Finnish Autism Spectrum Screening Questionnaire (ASSQ) for clinical settings and total population screening. J Autism Dev Disord 42:2162–2180. 10.1007/s10803-012-1464-5 22461223 10.1007/s10803-012-1464-5

[30] Orgiles M, Fernandez-Martinez I, Guillen-Riquelme A et al (2016) A systematic review of the factor structure and reliability of the Spence Children’s Anxiety Scale. J Affect Disord 190:333–340. 10.1016/j.jad.2015.09.055 26544617 10.1016/j.jad.2015.09.055

[31] Pavuluri MN, Henry DB, Carbray JA et al (2004) Open-label prospective trial of risperidone in combination with lithium or divalproex sodium in pediatric mania. J Affect Disord 82(Suppl 1):S103–S111. 10.1016/j.jad.2004.05.017 15571784 10.1016/j.jad.2004.05.017

[32] Jakobsson J, Julin AL, Persson G, Malm C (2021) Darwinian Selection Discriminates Young Athletes: the Relative Age Effect in Relation to Sporting Performance. Sports Med Open 7:16. 10.1186/s40798-021-00300-2 33650038 10.1186/s40798-021-00300-2 PMC7921243

[33] Kelleher I, Harley M, Murtagh A, Cannon M (2011) Are screening instruments valid for psychotic-like experiences? A validation study of screening questions for psychotic-like experiences using in-depth clinical interview. Schizophr Bull 37:362–369. 10.1093/schbul/sbp057 19542527 10.1093/schbul/sbp057 PMC3044617

[34] Shaffer D, Gould MS, Brasic J et al (1983) A children’s global assessment scale (CGAS). Arch Gen Psychiatry 40:1228–1231. 10.1001/archpsyc.1983.01790100074010 6639293 10.1001/archpsyc.1983.01790100074010

[35] Bzdok D, Meyer-Lindenberg A (2018) Machine Learning for Precision Psychiatry: Opportunities and Challenges. Biol Psychiatry Cogn Neurosci Neuroimaging 3:223–230. 10.1016/j.bpsc.2017.11.007 29486863 10.1016/j.bpsc.2017.11.007

[36] Newcorn JH, Sutton VK, Weiss MD, Sumner CR (2009) Clinical responses to atomoxetine in attention-deficit/hyperactivity disorder: the Integrated Data Exploratory Analysis (IDEA) study. J Am Acad Child Adolesc Psychiatry 48:511–518. 10.1097/CHI.0b013e31819c55b2 19318988 10.1097/CHI.0b013e31819c55b2

[37] McVey Neufeld SF, Ahn M, Kunze WA, McVey Neufeld KA (2024) Adolescence, the Microbiota-Gut-Brain Axis, and the Emergence of Psychiatric Disorders. Biol Psychiatry 95:310–318. 10.1016/j.biopsych.2023.10.006 37839790 10.1016/j.biopsych.2023.10.006

[38] Whitely M, Raven M, Timimi S et al (2019) Attention deficit hyperactivity disorder late birthdate effect common in both high and low prescribing international jurisdictions: a systematic review. J Child Psychol Psychiatry 60:380–391. 10.1111/jcpp.12991 30317644 10.1111/jcpp.12991 PMC7379308

[39] Caye A, Petresco S, de Barros AJD et al (2020) Relative Age and Attention-Deficit/Hyperactivity Disorder: Data From Three Epidemiological Cohorts and a Meta-analysis. J Am Acad Child Adolesc Psychiatry 59:990–997. 10.1016/j.jaac.2019.07.939 31442562 10.1016/j.jaac.2019.07.939

[40] Holland J, Sayal K (2019) Relative age and ADHD symptoms, diagnosis and medication: a systematic review. Eur Child Adolesc Psychiatry 28:1417–1429. 10.1007/s00787-018-1229-6 30293121 10.1007/s00787-018-1229-6 PMC6800871

[41] Kim JW, Sharma V, Ryan ND (2015) Predicting Methylphenidate Response in ADHD Using Machine Learning Approaches. Int J Neuropsychopharmacol 18:pyv052. 10.1093/ijnp/pyv052 10.1093/ijnp/pyv052 PMC4756719 25964505

[42] Farhat LC, Flores JM, Behling E et al (2022) The effects of stimulant dose and dosing strategy on treatment outcomes in attention-deficit/hyperactivity disorder in children and adolescents: a meta-analysis. Mol Psychiatry. 10.1038/s41380-021-01391-9 35027679 10.1038/s41380-021-01391-9

[43] Adamo N, Seth S, Coghill D (2015) Pharmacological treatment of attention-deficit/hyperactivity disorder: assessing outcomes. Expert Rev Clin Pharmacol 8:383–397. 10.1586/17512433.2015.1050379 26109097 10.1586/17512433.2015.1050379

[44] Lugo-Candelas C, Corbeil T, Wall M et al (2021) ADHD and risk for subsequent adverse childhood experiences: understanding the cycle of adversity. J Child Psychol Psychiatry 62:971–978. 10.1111/jcpp.13352 33289088 10.1111/jcpp.13352 PMC8169708

[45] Michelini G, Lenartowicz A, Vera JD et al (2023) Electrophysiological and Clinical Predictors of Methylphenidate, Guanfacine, and Combined Treatment Outcomes in Children With Attention-Deficit/Hyperactivity Disorder. J Am Acad Child Adolesc Psychiatry 62:415–426. 10.1016/j.jaac.2022.08.001 35963559 10.1016/j.jaac.2022.08.001 PMC9911553

[46] Myer NM, Boland JR, Faraone SV (2018) Pharmacogenetics predictors of methylphenidate efficacy in childhood ADHD. Mol Psychiatry 23:1929–1936. 10.1038/mp.2017.234 29230023 10.1038/mp.2017.234 PMC7039663

