# Supplementary material for: Can response to ADHD medication be predicted?
Source: Eur Child Adolesc Psychiatry. 2025 Jan 29;34(8):2431–42. doi: 10.1007/s00787-025-02650-8 (PMC12397174; doi:10.1007/s00787-025-02650-8)
Supplement: Supplementary file 1 — Supplementary file1 (DOCX 28 KB) [file 787_2025_2650_MOESM1_ESM.docx]

Supplementary Table S1

Can response to ADHD medication be predicted?

Maria M. Lilja^1^, Paul Lichtenstein^2^, Eva Serlachius^3,4^ Jyoti Bhagia^5^, Kerstin Malmberg^6^, Christer Malm^7^, Fabian Lenhard^4^ Linda Halldner^1,2^

1Department of Clinical Sciences, Child and Adolescent Psychiatry, Umea University, Umea, Sweden. 2Department of Medical Epidemiology and Biostatistics, Karolinska Institutet, Stockholm, Sweden. ^3^Department of Clinical Sciences, Faculty of Medicine, Section of Child and Adolescent Psychiatry, Lund University, Lund, Sweden. ^4^Centre for Psychiatry Research, Department of Clinical Neuroscience, Karolinska Institutet, Stockholm, Sweden. ^5^Department of Psychiatry and Psychology, Mayo Clinic, Rochester, MN, USA. ^6^Centre for Psychiatry Research, Department of Child and Adolescent Research Center, Stockholm, Sweden. Affiliated to the Department of Clinical Neuroscience. ^7^Department of Community Medicine and Rehabilitation, Section of Sports Medicine, Umeå School of Sport Sciences, Umea University, Umea, Sweden.

Corresponding author:

Maria M. Lilja

[Maria.lilja@umu.se](mailto:Maria.lilja@umu.se)

Supplementary Table S1. The National Quality Register, BUSA

BUSA is a Swedish National Quality Register for monitoring and evaluating the treatment outcomes of confirmed ADHD diagnoses in children and adolescents. Included diagnoses are ICD F90.0-F90.9, and in 2015 the additional national diagnoses ICD F90.0A, F90.0B, F90.0C, and F90.0X, were added.

The BUSA register contains data from 2004. In 2016 significant modifications were implemented in the BUSA register to enhance its reliability and validity. Thus from 2016 BUSA was named BUSA 2.0.

BUSA encompasses a wide range of variables, including school type, psychosocial problems, neuropsychiatric disabilities, alcohol and drug use screening, physical and mental co-morbidities, self-harm, suicide attempts, nicotine use, inter-agency collaboration, non-pharmacological interventions, cognitive ability, as well as pharmacological treatment, and adverse drug reactions. The SNAP-IV questionnaire is also included.

For a comprehensive and exhaustive list of all variables, please refer to the registry holder below.

BUSA closed for registration on June 30, 2021, due to ceased funding. Data is currently archived within the Stockholm County healthcare area (SLSO) at the Center for Psychiatry Research. Data extraction for research purposes is administered by the Research, Development, and Education Support Unit at SLSO, Region Stockholm: [fouu.slso@regionstockholm.se](mailto:fouu.slso@regionstockholm.se).
