# Supplementary material for: Can response to ADHD medication be predicted?
Source: Eur Child Adolesc Psychiatry. 2025 Jan 29;34(8):2431–42. doi: 10.1007/s00787-025-02650-8 (PMC12397174; doi:10.1007/s00787-025-02650-8)
Supplement: Supplementary file 2 — Supplementary file2 (DOCX 34 KB) [file 787_2025_2650_MOESM2_ESM.docx]

Supplementary Tables S2 and S3

Can response to ADHD medication be predicted?

Maria M. Lilja^1^, Paul Lichtenstein^2^, Eva Serlachius^3,4^ Jyoti Bhagia^5^, Kerstin Malmberg^6^, Christer Malm^7^, Fabian Lenhard^4^ Linda Halldner^1,2^

1Department of Clinical Sciences, Child and Adolescent Psychiatry, Umea University, Umea, Sweden. 2Department of Medical Epidemiology and Biostatistics, Karolinska Institutet, Stockholm, Sweden. ^3^Department of Clinical Sciences, Faculty of Medicine, Section of Child and Adolescent Psychiatry, Lund University, Lund, Sweden. ^4^Centre for Psychiatry Research, Department of Clinical Neuroscience, Karolinska Institutet, Stockholm, Sweden. ^5^Department of Psychiatry and Psychology, Mayo Clinic, Rochester, MN, USA. ^6^Centre for Psychiatry Research, Department of Child and Adolescent Research Center, Stockholm, Sweden. Affiliated to the Department of Clinical Neuroscience. ^7^Department of Community Medicine and Rehabilitation, Section of Sports Medicine, Umeå School of Sport Sciences, Umea University, Umea, Sweden.

Corresponding author:

Maria M. Lilja

[Maria.lilja@umu.se](mailto:Maria.lilja@umu.se)

Supplementary Table S2. Summary of all measurements

| **Category** | **Variable** | **Unit/definition** | **Format** |
| --- | --- | --- | --- |
| Outcome | SNAP-IV score reduction | Responders (≥40% reduction), Intermediate responders (<40% but ≥20%), Non-responders (<20%) | categories |
| Anthropometrics | Body weight | Kg | continuous |
|  | Body Height | Cm | continuous |
|  | Systolic blood pressure | mm Hg | continuous |
|  | Diastolic blood pressure | mm Hg | continuous |
|  | Heart rate | Beats per minute | continuous |
|  |  |  |  |
| Personal details | Age at baseline | Year | continuous |
|  | Relative age | Tertiles: January-April, May-August, September-December | categories |
|  | Region | Stockholm, Umeå, Gotland | categories |
|  | Sex | Male, female | dichotomized |
|  |  |  |  |
| Pharmacological treatment details | Initiation month | Tertiles: January-April, May-August, September-December | categories |
|  | ADHD medication, including methylphenidate, dexamfetamine, lisdexamfetamine, atomoxetine, guanfacine, or combinations thereof | Yes or No | dichotomized |
|  |  |  |  |
| Other | IQ | Above average IQ ≥ 108, Average IQ 93-107, Below average IQ ≤ 92, Difficult to access. | four categories |
|  | psychotic-like experiences | we used a separate single question: Have you ever heard voices or sounds that nobody else can hear? The answer ‘yes definitely’ or ‘maybe’, was considered as a psychotic-like experience and registered as hallucination symptoms. The answer ‘no’ was defined as the absence of psychotic-like experiences | dichotomized |
| Questionnaires | SNAP-IV: Total score (items 1-30) | Not at all = 0, Just a little =1, Quite a bit = 2, Very much =3 | continuous |
|  | SNAP-IV: Oppositional Defiant Disorder symptoms (items 11-19) | Not at all = 0, Just a little =1, Quite a bit = 2, Very much =3 | continuous |
|  | SNAP-IV: predominantly inattentive symptoms (items 1-9) | Not at all = 0, Just a little =1, Quite a bit = 2, Very much =3 | continuous |
|  | SNAP-IV: predominantly hyperactive-impulsive symptoms (items 11-19) | Not at all = 0, Just a little =1, Quite a bit = 2, Very much =3 | continuous |
|  | SNAP-IV: combination of predominantly hyperactive-impulsive and inattentive symptoms (items 1-9 and items 11-19) | Not at all = 0, Just a little =1, Quite a bit = 2, Very much =3 | continuous |
|  | ASSQ (27 items) | No = 0, Somewhat =1, Yes =2 | continuous |
|  | SCAS-P (39 items. No. 1-38 numeric. No. 39 is a string variable, not used). | Never = 0, Sometimes = 1, Often = 2, Always =3 | continuous |
|  | P-SEC (50 items split into 11 categories*. No. 1-49 numeric. No.50 string variable, not used). | None = 0, Mild/sometimes but tolerable = 1, Moderate/interferes somewhat =2, Severe/interferes a lot = 3. | continuous |
|  | CGAS score (Scores between 1-100 categorized in: 1-10, 11-20, 21-30, 31-40, 41-50, 51-60, 61-70, 71-80, 81-90, 91-100). | Unable to function in almost all areas (21-30).  Major impairment in functioning in several areas, and unable to function in one or these areas (31-40).  Moderate degree of interference in functioning in most social areas or severe impairment of functioning in one area (41-50).  Variable functioning with sporadic difficulties or symptoms in several but not all social areas (51-60).  Some difficulty in a single area, but generally functioning pretty well (61-70). | five categories |

*The gastrointestinal system, the central nervous system, the endocrine system, mood/behavioral changes, the cardiovascular system, the immune system, the skin, the renal system, sexual concerns/problems, allergic reactions and other symptoms.

Supplementary Table S3. Summary of Statistical Analyses

| **Order** | **Aim** | **Statistical procedures and variables involved** |
| --- | --- | --- |
| 1 | Test for differences in baseline characteristics between our outcome groups | Kruskal-Wallis one-way analysis of variance: ASSQ, SNAP-IV, P-SEC, and SCAS.  One-way ANOVA tests: heart rate, systolic and diastolic blood pressure, body weight, and height, and age at baseline.  Chi-Square test of independence: tertiles of birth, tertiles of pharmacological treatment initiation month, regions, pharmacological treatment, psychotic-like experiences, sex, IQ, and CGAS |
| 1 | Test for differences between individuals with a completely missing SNAP-IV scale at baseline and/or at follow-up | Kruskal-Wallis one-way analysis of variance: ASSQ, SNAP-IV, P-SEC, and SCAS.  One-way ANOVA tests: heart rate, systolic and diastolic blood pressure, body weight, and height, and age at baseline.  Chi-Square test of independence: tertiles of birth, tertiles of pharmacological treatment initiation month, regions, pharmacological treatment, psychotic-like ecperiences, sex, IQ, and CGAS |
| 2 | Comparing the three outcome groups (predictive factors) | Unadjusted Multinomial logistic regression for all 22 independent variables |
| 3 | Comparing the three outcome groups (predictive factors). | Machine learning. First comparing the performance of seven different machine learning methods. The machine learning model with the highest R2 was chosen for further analysis (Bootstrap Forest). Second, the 13 selected predictors were ranked by their contribution to the bootstrap forest model and entered into the model. Models were created using 75% of the data for training and 25% for validation. |
| 4 | Subgroup analysis to assess if ADHD medication substances differed between the outcome groups | Chi2 tests. |
| 5 | Sensitivity analyses of the cohort in the Västerbotten Region | Procedures 2-3 were repeated for only the Västerbotten Region. |
